# Functional divergence of the gut microbiome associated with lifestyle and helminth infection in Indigenous Peninsular Malaysian

**DOI:** 10.21203/rs.3.rs-7706316/v1

**Published:** 2026-01-23

**Authors:** Soo Ching Lee, Mian Zi Tee, Zeyang Shen, Yi Xian Er, Redekar Neelam, Ken Cadwell, Julia A Segre, Yvonne Ai Lian Lim, P’ng Loke

**Affiliations:** 1 Type 2 Immunity Section, Laboratory of Parasitic Diseases, National Institute of Allergy and Infectious Diseases, National Institute of Health, Bethesda, MD, USA.; 2 Department of Parasitology, Faculty of Medicine, Universiti Malaya, KL, Malaysia.; 3 Microbial Genomics Section, Translational and Functional Genomics Branch, National Human Genome Research Institute, National Institutes of Health, Bethesda, MD, USA.; 4 School of Molecular Biosciences, College of Veterinary Medicine, Washington State University, Pullman, WA, USA.; 5 Integrated Data Sciences Section, Research Technologies Branch, National Institute of Allergy and Infectious Diseases, National Institutes of Health, Bethesda, MD, USA.; 6 Division of Gastroenterology and Hepatology, Department of Medicine, University of Pennsylvania Perelman School of Medicine, Philadelphia, PA, USA.; 7 Department of Pathobiology, University of Pennsylvania Perelman School of Veterinary Medicine, Philadelphia, PA, USA.

## Abstract

Gut microbiome catalogs from Indigenous Southeast Asian populations remain underrepresented. Here, we integrated metagenomic and metatranscriptomic data from Indigenous Orang Asli (OA) in Peninsular Malaysia and urban residents of Kuala Lumpur (KL), together with immune profiling, to investigate gut microbial activity and functions associated with lifestyle and helminth infection. *Prevotella* showed significantly higher transcriptional activity in OA, whereas *Bacteroides* was more active in KL, corresponding to distinct immune signatures. Microbial genome-wide association studies (mGWAS) revealed *Prevotella copri_A* variants were linked to lifestyle and host immunity, while *Blautia* strain variation was associated with helminth infection. Malaysian metagenome-assembled genomes (MAGs) uncovered 307 novel species, predominantly within Clostridia. Among these, the novel *HGM13006* species were enriched with genes for starch and sucrose metabolism, and the novel *Ruminococcus_D* species in flagellar assembly and chemotaxis. Together, these findings provide function-level insights into gut microbiome variation associated with lifestyle and helminth infection in an indigenous population.

The Orang Asli (OA) are the Indigenous peoples of Peninsular Malaysia, representing approximately 0.7% of the country’s population^1^. As the region earliest known settlers, OA communities have traditionally engaged in hunting, subsistence agriculture, and fishing. While some communities have gradually integrated into modern lifestyles, some who remain in the interior areas still experience limited access to basic health services. These disparities have contributed to a continued vulnerability to both communicable and non-communicable diseases^2^, including soil-transmitted helminth (STH) infections^3–6^, skin diseases^7–10^, malaria^6,11–13^, malnutrition^14–17^, obesity^18^, hypertension^18^ and other neglected conditions^19^. Among these, helminth infections are prevalent, affecting over 50%^5,6,20–22^ in this population and contributing to significant morbidity, such as anemia, stunted growth, impaired cognitive development in children, and reduced work productivity in adults^2,14,23,24^.

Over the past decade, our research team has been at the forefront of investigating the gut and skin microbiome of OA communities^3,4,8,25–27^. We have observed that helminth-infected OA individuals consistently exhibit higher gut microbial diversity than the uninfected^3,4^ and the helminths-gut microbiome interactions are context dependent across village^3^. In our recent shotgun metagenomic study of stool samples from five OA villages and urban Kuala Lumpur (KL) residents, we revealed a significant portion of the OA gut microbiome remains uncharacterized compared to KL^3^. While advances in metagenome-assembled genomes (MAGs) has significantly expanded microbiome reference catalogs, most collections were dominated by samples from Westernized societies^28–32^, with minimal representation from Asian^33^, African^34,35^, and Southeast Asian population^36^. To date, the only publicly available Southeast Asian gut metagenomes originate from urban Singapore^36^. Indigenous population, such as the OA remain underrepresented. Given their close interactions with the natural environments and reliance on diets derived from locally available resources, characterizing the OA gut microbiome offers a unique opportunity to explore how traditional ecological lifestyle and environmental exposure shape the gut microbiome. Expanding gut microbiome catalogs to include underserved populations is crucial for a more comprehensive understanding of global microbial diversity.

Much of the research on human gut microbiomes has focused on their taxonomic composition and genomic potential^3,4,28–36^, while the transcriptional activity of microbes, which reflects real-time functional output, remains underexplored. Urbanization has been shown to shape gut microbiota composition, often leading to reduced microbial diversity and shifts in key taxa^37–42^ such as *Prevotella* and *Bacteroides*. These two genera represent hallmarks of traditional, plant-based diets versus industrialized animal based-diets^37,39,41–48^. Shift in their balance (*Prevotella*/*Bacteroides* ratio) has been linked to metabolic disorders including obesity^49^, weight loss^42,50,51^ and diabetes^52^. In our previous 16S rRNA study, *Prevotella* was significantly more abundant in the OA community, while *Bacteroides* dominated in urban KL residents^27^. While many studies have assessed these genera abundance using 16S rRNA and shotgun metagenomic^39,40,43–46,48,53^, only recently have *Prevotella* genomes and their functions been studied across Western and non-Western cohorts^54^. However, their transcriptional activity, especially in helminth-endemic populations, remain largely unknown. Metatranscriptomic approaches, which measure real-time gene expression *in vivo*, provide critical insights into microbial function and host-microbe interactions. Applying this to Indigenous communities like the OA may reveal mechanisms through which lifestyle and helminth exposures modulate gut microbial function and host health.

In parallel, there has been growing interest in understanding the nucleotide-level diversity within bacterial communities. Similar to how human single-nucleotide polymorphisms (SNPs) influence health traits such as obesity^55^, type 2 diabetes^55^, cancer^56,57^, inflammatory bowel disease^58,59^ and weight loss^60^, SNPs within microbial genomes can significantly influence bacterial functions^61–64^, with potential implications for human health. Previous research has identified vast number of microbial SNPs^65,66^, some of which influences microbial drug metabolism^67^, potentially influence host drug responses or correlate with host phenotypes like BMI^68^. However, little is known about bacterial SNPs in relation to lifestyle factors and helminth infections, particularly in underrepresented population such as OA, where these infections are more prevalent.

This study used a combination of metagenomic and metatranscriptomic datasets to study gut microbiome and its function in five OA villages and urban KL residents. We assembled 15,867 MAGs from 650 stool metagenomes^3^. By integrating these MAGs with existing microbial genome catalogs, including the Unified Human Gastrointestinal Genome (UHGG) and genomes from Korean, Indian, and Japanese (KIJ) populations, we identified microbial species and functions specific to the Malaysian population. We also conducted metatranscriptomic analysis on 66 OA and 30 KL samples, representing one of the largest stools metatranscriptomic datasets generated from Southeast Asian Indigenous populations. Using the metatranscriptomic data, we deepened our understanding of the transcriptional activity of *Prevotella* and *Bacteroides* in different lifestyles and helminth-endemic populations, areas previously underexplored. Additionally, we integrated microbial SNPs and transcriptional data with host cytokine and chemokine measurements to explore how strain-level variation and microbial activity associates with host immune phenotypes.

## Results

### Gut prokaryotes specific to Malaysian based on metagenome assembled genomes (MAGs)

**Extended Data Fig. 1:**
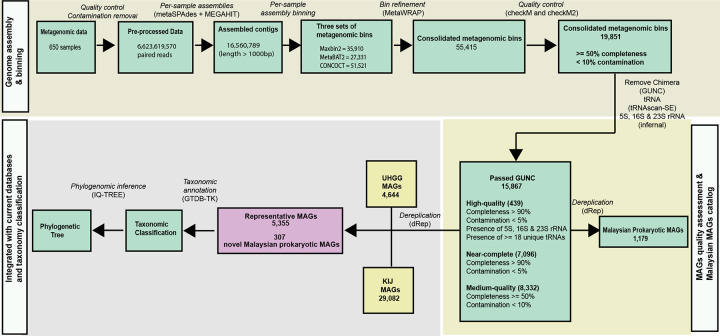
Overview of computational workflow and data outputs. Summary of the computational workflow, tools used, and primary data generated starting from genome assembly and binning, MAG quality assessment, integration between Malaysian, UHGG and KIJ databases, and taxonomic classification based on shotgun metagenomic (DNA) data.

**Fig. 1:**
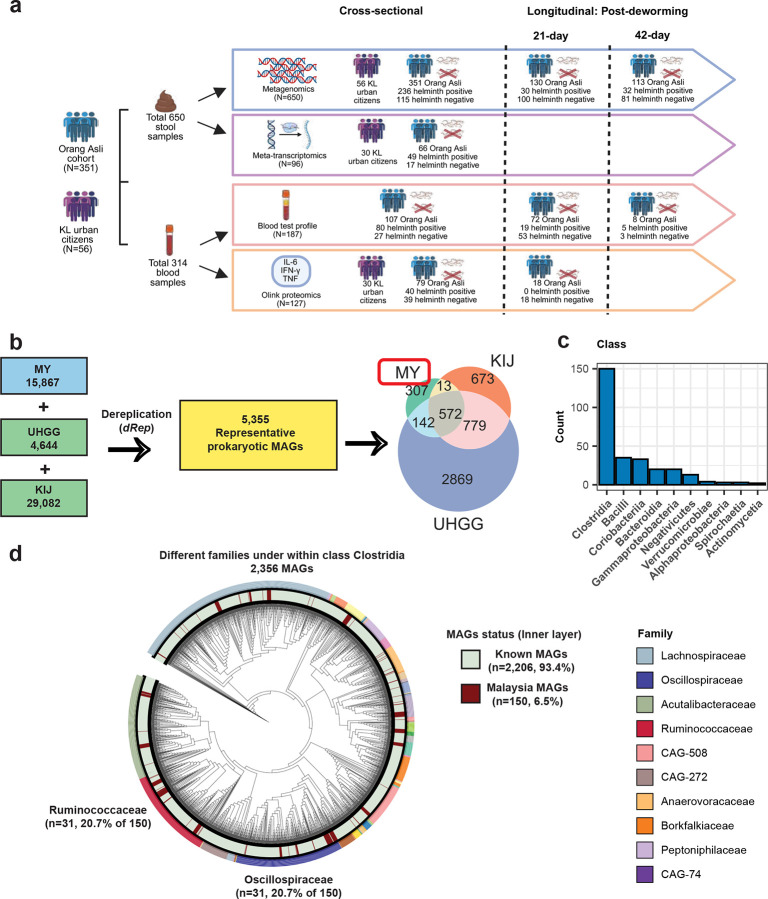
Gut metagenome assembled genomes (MAGs) specific to Malaysian. (**a**) Overview of the collected samples from Malaysian population, encompassing stool shotgun metagenomic (DNA) and metatranscriptomic (RNA) sequencing across cross-sectional and longitudinal collections at 21 and 42 days post-deworming. Helminth infection status is shown as positive (without “X”) and negative (marked with “X”). Blood samples were obtained from the same participants, with subsets processed for clinical serum testing and Olink proteomics (cytokines and chemokines). (**b**) Summary of 5,355 dereplicated stool prokaryotic MAGs from Malaysian, Unified Human Gastrointestinal Genome (UHGG) and Korean, Indian, Japanese (KIJ) datasets. The Venn diagram demonstrates the shared and unique gut prokaryotic (bacterial & archaeal) MAGs. 307 (5.7%) specific to Malaysia, 572 (10.7%) are shared among the three databases, 673 (12.6%) specific to KIJ, and 2,869 (53.6%) specific to UHGG. (**c**) Bar plot displaying the taxonomic classification at the class level for the prevalent Malaysian bacterial MAGs. (**d**) Phylogenetic tree showing the Malaysian bacterial MAGs and known MAGs within class Clostridia, annotated using GTDB taxonomy.

We previously reported metagenomic sequencing and reference-based analysis of 650 stool samples collected from 351 OA individuals across five villages, both cross-sectionally and longitudinally, as well as 56 urban residents from KL^3^. To characterize the large fraction of unmapped reads, we assembled this metagenomic dataset to generate 15,867 chimeric- and quality-checked metagenomic assembled genomes (MAGs) with completeness > 50%, contamination < 10%) (Fig. 1a and 1b, Extended Data Fig. 1 and Supplementary Table 1). By integrating the 15,867 MAGs with the UHGG (n = 4,644)^30^ and KIJ (n = 29,082)^33^ MAGs, and dereplicating genomes to select only those with <95% average nucleotide identity, we generated an expanded catalog containing 5,355 prokaryotic species (Fig. 1b). Notably, 307 (5.7% of 5,355) of the prokaryotic species were specific to the Malaysian dataset, 572 (10.7%) are shared among the three databases, 673 (12.6%) specific to KIJ^33^, and 2,869 (53.6%) specific to UHGG^30^ (Fig. 1b).

Of these 5,355 prokaryotic species, 5,327 were classified as bacteria, including 291 that were Malaysia-specific, and 28 as archaea, of which 16 were Malaysia-specific (Supplementary Table 2). While the Malaysia-specific bacterial species were identified within various phyla, they predominantly belonged to the Clostridia class (n = 150, 51.5% of 291) (Fig. 1c). Within the Clostridia class, a significant number of species were classified under the families Ruminococcaceae (31, 20.7% of 150) and Oscillospiraceae (31, 20.7% of 150) (Fig. 1d).

### Microbial functions of Malaysian-specific Clostridia species

**Extended Data Fig. 2:**
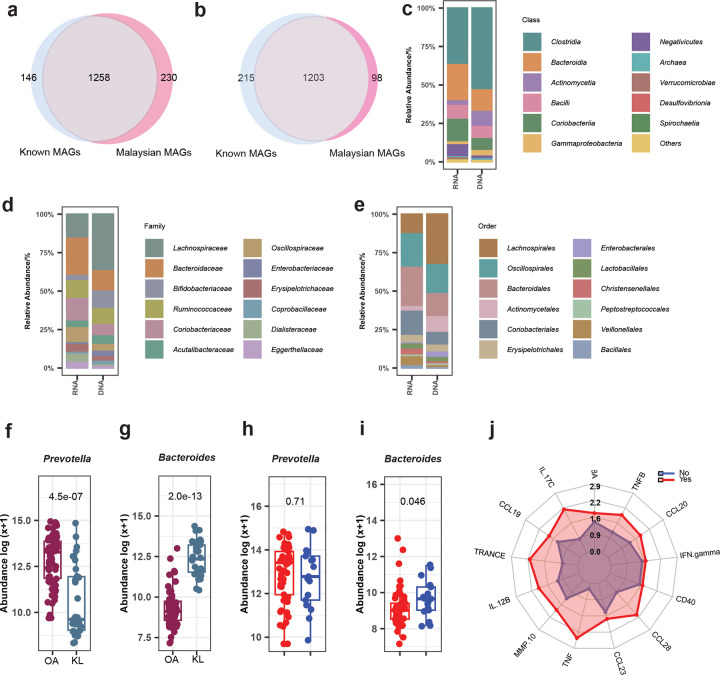
Comparison of enrichment pathway, taxonomic and host immune profiles between groups. Venn diagram of KEGG gene IDs comparing between Malaysian and known MAGs for (**a**) *HGM13006* and (**b**) *Ruminococcus_D*. Bar plots displaying the relative abundance of the top taxa at the (**c**) class, (**d**) family and (**e**) order levels, comparing RNA and DNA data. Boxplots showing metagenomic abundance of (**f**, **h**) *Prevotella* and (**g**, **i**) *Bacteroides* between OA and KL groups and *Bacteroides* between helminth infection. (**j**) Radar plot displaying log-transformed median levels of 13 inflammatory cytokines and chemokines significantly different between helminth infected and uninfected individuals.

**Fig. 2:**
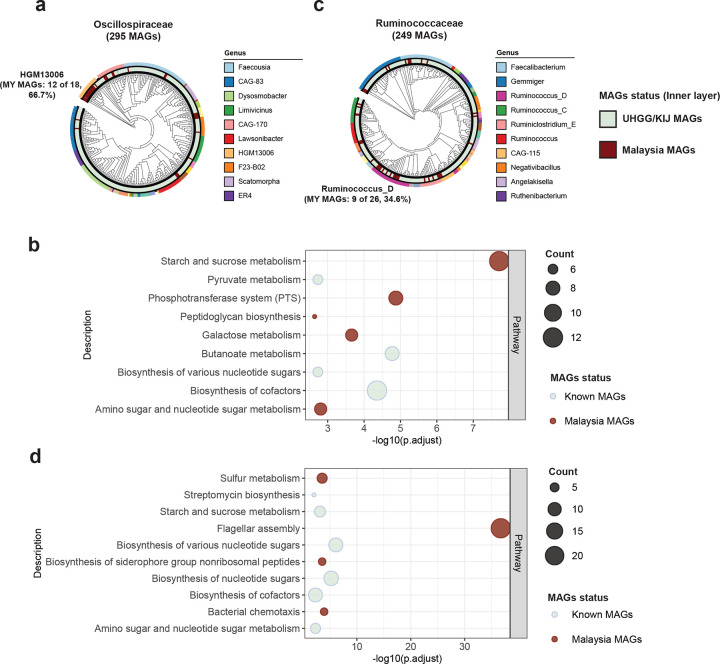
Malaysian MAGs under class Clostridia and their enrichment pathways. Phylogenetic tree of the Malaysian and known bacterial MAGs annotated with GTDB taxonomy within the family (**a**) Oscillospiraceae and (**c**) Ruminococcaceae. Dot plots show the Functional pathways enriched in Malaysian MAGs compared to known MAGs for (**b**) *HGM13006* and (**d**) *Ruminococcus_D*. Dot color indicates the MAG origin (Malaysian: brown; known: light green), dot size corresponds to the number of KEGG orthologs annotated to each pathway and x- axis represents the statistical significance of pathway enrichment as −log_10_ (adjusted p-value).

Within the family Oscillospiraceae, we identified 18 species belonging to the genus *HGM13006*, including 12 novel species specific to Malaysian (Fig. 2a). This genus remains relatively understudied, with limited information available. A comparison of gene contents between 12 novel Malaysian species and 6 known species of this genus revealed 230 genes unique to the novel species, 1,258 shared genes, and 146 genes unique to the known species (Extended Data Fig. 2a). Functional analysis of the 230 Malaysian-specific genes revealed significant enrichment in pathways related to starch and sucrose metabolism and the phosphotransferase system (PTS) (Fig. 2b; Supplementary Table 3), indicating a potential functional role of the novel *HGM13006* species in carbohydrate metabolism.

In the family Ruminococcaceae, we observed 26 species within the genus *Ruminococcus_D*, including 9 novel Malaysian species (Fig. 2c). Comparison between novel and known species identified 98 genes specific to the novel species, 1,203 shared genes, and 215 genes specific to the known species (Extended Data Fig. 2b). In contrast to *HGM13006*, Malaysian-specific genes within the genus *Ruminococcus_D* showed enrichment in pathways related to flagellar assembly and bacterial chemotaxis (Fig. 2d; Supplementary Table 4), suggesting these genomes encode genetic traits associated with motility and environmental sensing.

### Transcriptional activity of *Prevotella* & *Bacteroides* in relation to lifestyle differences and helminth infection

**Fig. 3:**
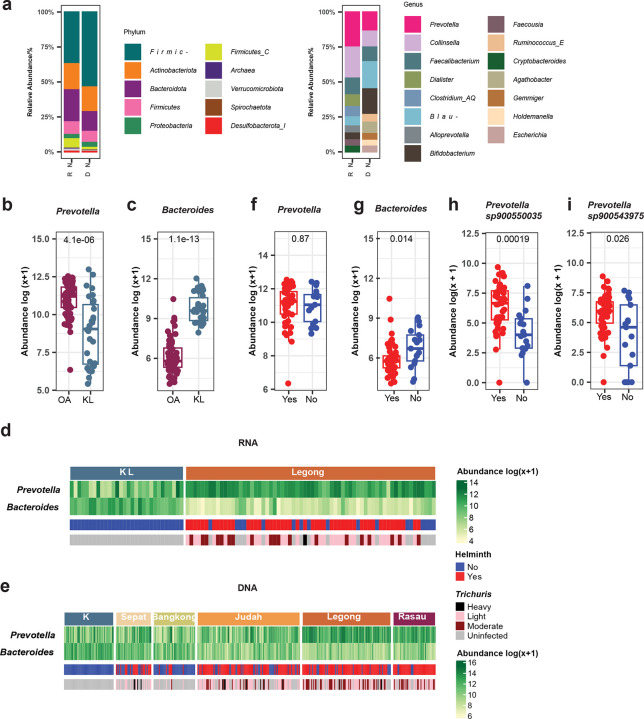
Transcriptional activity of *Prevotella* & *Bacteroides*. (**a**) Bar plots displaying the relative abundance of the top taxa at the phylum (left) and genus levels (right), comparing RNA and DNA data. Boxplots showing transcriptional activity of (**b**) *Prevotella* and (**c**) *Bacteroides* between OA and KL groups. Heatmaps of (**d**) transcriptional activity and (**e**) metagenomic abundance for *Prevotella* and *Bacteroides*, with samples in columns categorized by village. The first horizontal bar color indicates intestinal helminth infection status, while the second shows the *Trichuris* infection intensity (ranging from 12 to 119,875 eggs per gram, from light to heavy intensity). The boxplot illustrates the transcriptional activity of the genera (**f**) *Prevotella*, (**g**) *Bacteroides*, and (**h & i**) the Malaysian *Prevotella* species, comparing the differences between helminth infection statuses.

In addition to stool shotgun metagenomics, we sequenced 96 metatranscriptomic samples from 66 OA individuals and 30 urban residents from KL, Malaysia. To determine whether the gut bacteria with higher abundance are also transcriptionally active in the cohort, we first analyzed baseline samples collected prior to any helminth treatment. We mapped 407 pre-anthelminthic treatment metagenomic (DNA) data and 96 metatranscriptomic (RNA) data to an integrated database that includes our expanded MAG catalog (see Methods). We observed differences in the relative abundance of the top taxa between the two data types, which became more pronounced at finer taxonomic levels (Fig. 3a, Extended Data Fig. 2c–e). At the phylum level, *Firmicutes_A* was dominant in both datasets but showed a lower relative abundance in RNA (37.0% ± 13.5%) compared to DNA (53.7% ± 14.8%) samples, while *Bacteroidota* displayed higher transcriptional representation (24.1% ± 14.6%) relative to the DNA-based relative abundance (14.0% ± 12.3%) (Fig. 3a; Supplementary Table 5). At the genus level, *Prevotella* emerged as the most transcriptionally active taxon (mean RNA relative abundance :12.6% ± 13.3% vs mean DNA relative abundance: 7.2% ± 10.8%), followed by *Collinsella* (11.3% ± 9.2% vs 6.0% ± 3.9%), *Faecalibacterium* (6.0% ± 5.9% vs 5.0% ± 3.7%) and *Dialister* (4.2% ± 4.7% vs 0.7% ± 0.8%). Interestingly, *Dialister* was prominent in RNA but not among the top genera in DNA data. Conversely, *Blautia* which ranked highly in the DNA-based profiles, showed reduced representation in RNA (3.3% ± 2.9% vs 10.8% ± 6.6%) (Fig. 3a; Supplementary Table 6).

**Extended Data Fig. 3:**
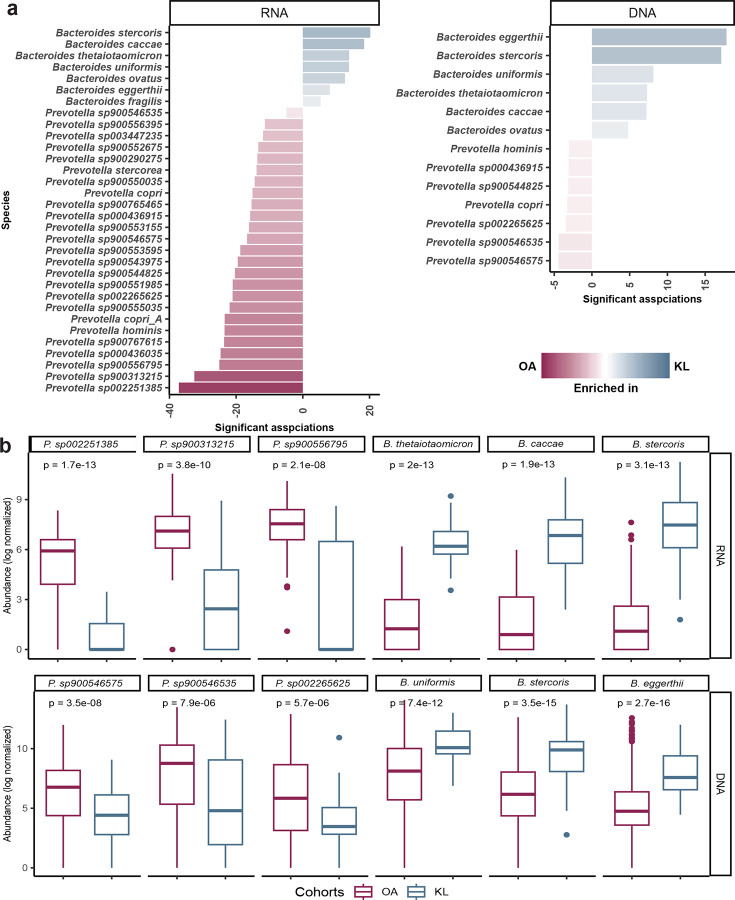
Differential activity and abundance of *Prevotella* and *Bacteroides* species between OA and KL populations. (**a**) Bar plots showing *Prevotella* and *Bacteroides* species with significantly different transcriptional activity (RNA; left) and genomic abundance (DNA; right) between OA and KL individuals. This were assessed using MaAsLin2, adjusting for age, gender, and helminth infection status. (**b**) Box plots displaying log-transformed abundance of selected top differentially active and abundant *Prevotella* and *Bacteroides* species between groups in both RNA (top) and DNA datasets (bottom).

*Prevotella* and *Bacteroides* are well-known biomarkers of lifestyle, exhibiting an inverse relationship, as seen in both metagenomic and 16S rRNA sequencing studies^37,39,41–48^. We compared matched RNA and DNA data from the same 96 individuals to evaluate transcriptional activity alongside taxonomic abundance. In line with the *Prevotella*-*Bacteroides* inverse lifestyle paradigm, we observed significantly higher transcriptionally activity of *Prevotella* in the OA cohort compared to the KL cohort (p= 4.1^e-6^) (Fig. 3b), while *Bacteroides* activity was higher in the KL cohort (p= 1.1^e-13^) (Fig. 3c). These findings mirrored trends in DNA abundance observed in the same cohort (Extended Data Fig. 2f & g). To statistically access the transcriptional differences at species level, we applied MaAsLin2^69^ on both RNA and DNA datasets, adjusting for age, gender and helminth infection status. This analysis identified 25 *Prevotella* species with adequate coverage (≥20% at the 90th percentile) including *P. sp002251385, P. sp900313215, P. sp90055679*5, and *P. sp000436035*, with significantly higher transcriptional activity in OA (Extended Data Fig. 3, Supplementary Table 7, 8). In contrast, 7 *Bacteroides* species, which include *B. stercoris*, *B. caccae*, *B. thetaiotaomicron* and *B. uniformis*, were more active in KL. Notably, no *Prevotella* species were more active in KL and no *Bacteroides* species were more active in OA. We observed a similar pattern at the DNA level with 7 *Prevotella* species and 6 *Bacteroides* species significantly different between groups. *Prevotella* species such as *P*. *sp900546575, P. sp900546535* and *P. sp002265625* were significantly more abundant in the OA cohort. Conversely, *Bacteroides* species including *B. eggerthii, B. stercoris* and *B. uniformis* were more abundant in KL (Extended Data Fig. 3 and Supplementary Table 7). These species demonstrated consistent differential patterns in both RNA and DNA, indicating robust lifestyle-associated differences.

We also examined the potential influence of helminth infection on both *Prevotella* and *Bacteroides* transcriptional activity. At the individual level, a heatmap revealed variation in RNA profiles linked to helminth infection status, particularly with *Trichuris* infection intensity (ranging from 12 to 119,875 eggs per gram, from light to heavy intensity) (Fig. 3d). Given that RNA data were available from only one village, we extended the analysis to DNA data across five villages with varying helminth prevalence. We noticed *Prevotella* abundance was higher in villages with high infection prevalence (e.g., Legong and Rasau), whereas *Bacteroides* was more abundant in villages with low infection prevalence (e.g., Sepat and Bangkong) (Fig. 3e). Focusing on the one village with RNA data, we compared the transcriptional activity between helminth-infected and uninfected individuals. *Bacteroides* displayed significantly higher transcriptional activity in uninfected individuals (p= 0.014), while *Prevotella* showed no significant difference (p= 0.870) (Fig. 3f and 3g). Interestingly, analysis of five Malaysian *Prevotella* species revealed that two species, *P. sp900543975* (p= 0.026) and *P. sp900550035* (p= 0.0019) were significantly more transcriptionally active in helminth-infected individuals than in uninfected individuals, indicating species-specific variation in response to helminth infection (Fig. 3h & i, Extended Data Fig. 2h & i).

**Extended Data Fig. 4:**
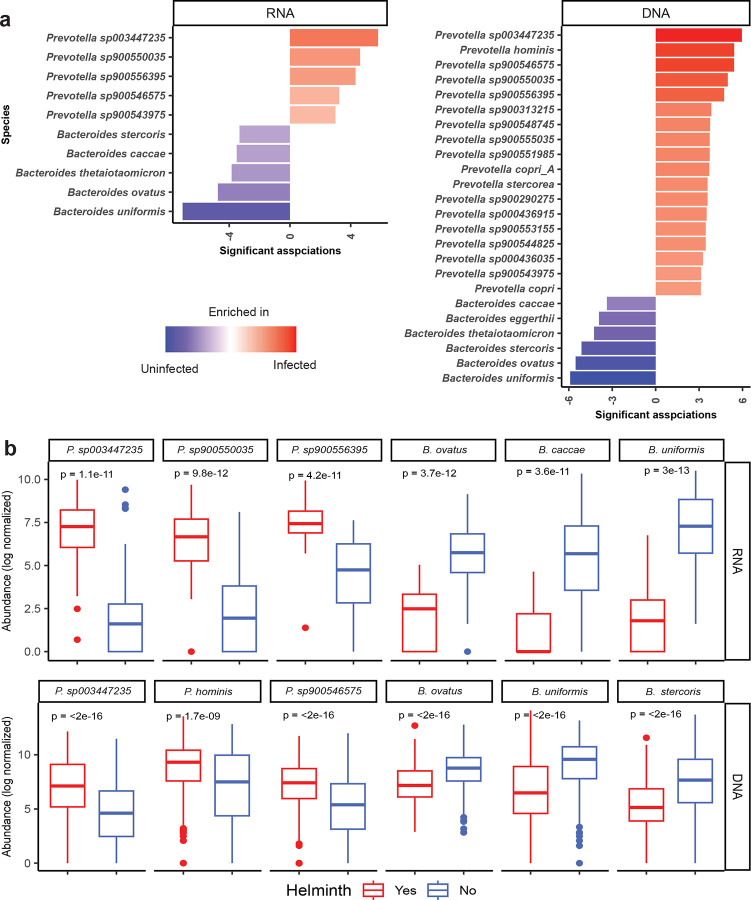
Differential activity and abundance of *Prevotella* and *Bacteroides* species between helminth infected and uninfected OA populations. (**a**) Bar plots showing *Prevotella* and *Bacteroides* species with significantly different transcriptional activity (RNA; left) and genomic abundance (DNA; right) between OA and KL individuals. This were assessed using MaAsLin2, adjusting for age, gender, and helminth infection status. (**b**) Box plots displaying log-transformed abundance of selected top differentially active and abundant *Prevotella* and *Bacteroides* species between groups in both RNA (top) and DNA datasets (bottom).

To further assess the association between helminth infection and *Prevotella* and *Bacteroides*, we performed differential abundance analysis on the RNA and DNA data from the same village, adjusting for age and gender. Result revealed that five *Prevotella* species including the two Malaysian *Prevotella* MAGs noted above (*P. sp900543975* and *P. sp900550035*) along with *P. sp003447235 and P. sp900546575* were significantly enriched in both transcriptionally activity and genomic abundance in helminth-infected individuals (Extended Data Fig. 4 and Supplementary Table 9). In contrast, five *Bacteroides* species, *B. uniformis*, *B. caccae*, *B. thetaiotaomicron, B. ovatus* and *Bacteroides stercoris* were significantly reduced in both abundance and transcriptional activity among infected individuals (Extended Data Fig. 4 and Supplementary Table 9).

### Association between the *Prevotella*/*Bacteroides* ratio with host immune responses

**Fig. 4:**
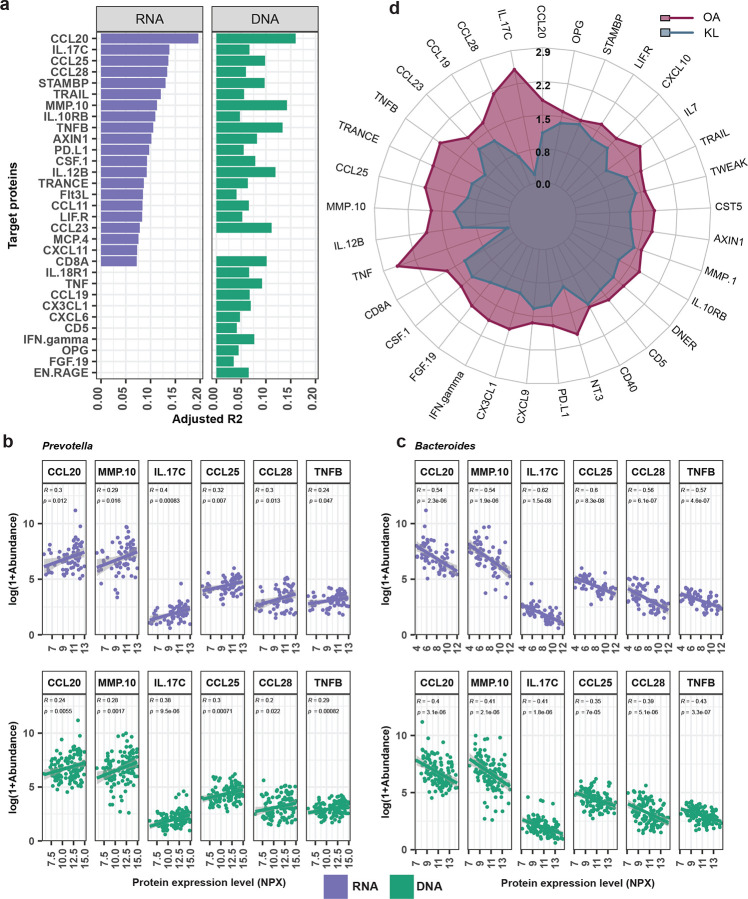
Association between the *Prevotella*/*Bacteroides* ratio with host immune responses. (**a**) Horizontal barplots showing cytokines and chemokines significantly associated with the *Prevotella*/*Bacteroides* ratio, identified using a simple linear regression model based on 127 DNA and 68 RNA samples. The left panel displays 21 proteins from RNA samples and the right panel shows 29 proteins from DNA samples. Bars length represents the effect size as indicated by the adjusted R2. Representative scatter plots showing Spearman correlations between selected top proteins and log-transformed abundance (log[1+Abundance]) of (**b**) *Prevotella* and (**c**) *Bacteroides*. Correlation coefficients (R) and corresponding p-values are annotated. (**d**) Radar plot displaying the log-transformed median levels of 33 inflammatory cytokines and chemokines that were significantly different between the OA (n=66) and KL (n=30) cohorts.

To explore how the abundance and transcriptional activity of the *Prevotella and Bacteroides* are associated with human host immune responses, we conducted a simple linear model analysis of the *Prevotella*/*Bacteroides* ratio with 68 cytokines and chemokines from the Olink inflammatory panel. The analysis included data from 68 RNA and 127 DNA samples with matched Olink data. We found that the *Prevotella*/*Bacteroides* ratio were significantly associated with 21 and 29 cytokines and chemokines, respectively, with 19 of these shared between RNA and DNA analysis (Fig. 4a & Supplementary Table 10). These included CCL20, IL17C, CCL25, CCL28, and TNFB (Fig. 4a & Supplementary Table 10).

These findings were confirmed at the individual genus level, in which *Prevotella* transcription and abundance was positively correlated with these cytokines in both RNA and DNA data (Fig. 4b). On the other hand, *Bacteroides* transcription and abundance negatively correlated with these cytokines (Fig. 4c). We also examined the association of *Prevotella/Bacteroides* ratio in relation to various parameters from blood tests (Supplementary Table 11); however, no significant associations were observed.

To identify how these cytokines and chemokines are associated with different lifestyle and urbanization, we compared these immune markers between OA and KL urban residents. We found that 33 cytokines and chemokines were significantly different between groups, including those identified earlier (Fig. 4d and Supplementary Table 12). All these markers were significantly higher in the OA compared to KL cohort (Fig. 4d). We further assessed whether these immune markers were associated with helminth infection status. Comparison between helminth-infected and uninfected individuals revealed that 13 markers, including CCL20 (p=0.0003) and IL17C (p=0.0052), were significantly high in helminth-infected individuals (Extended Data Fig. 2j and Supplementary Table 13). These findings suggest that the activity of *Prevotella* and *Bacteroides* is closely linked to immune profiles, and that both lifestyle and helminth exposure may modulate these relationships.

### Strain-level variations of the gut microbiome in the Orang Asli

**Fig. 5:**
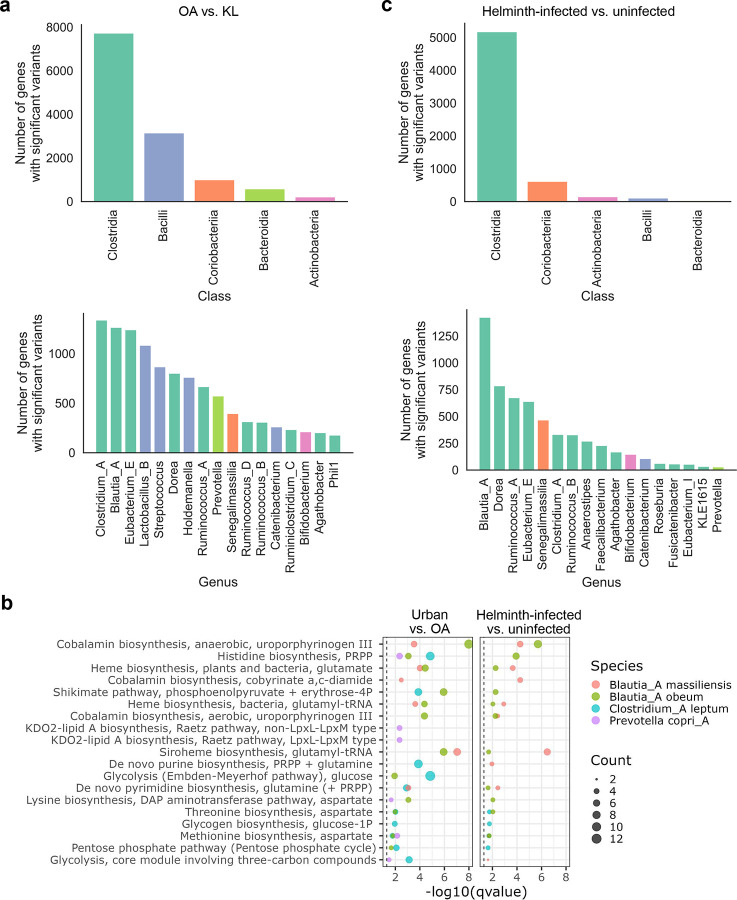
Strain-level variations in the gut microbiome associated with Orang Asli and helminth infection. (**a**) Number of genes harboring significant strain-level variants in the OA population compared to the KL cohort, grouped by taxonomic class (top) and genus (bottom). (**b**) KEGG modules enriched among genes with significant variants in representative species. Dot size indicates the number of genes per module; vertical lines denote statistical significance after correction for multiple testing. The top five enriched modules are shown for each species. (**c**) Number of genes with significant strain-level variants associated with helminth infection status, grouped by taxonomic class (top) and genus (bottom).

To gain deeper insight into strain-level genetic variation in the gut microbiome and its functional consequences in the OA, we applied a microbial genome-wide association study (mGWAS) approach to identify strain-level variants that differed between the OA and KL cohorts. We identified 84 species with at least one significant variant, with the taxa harboring the greatest number of significant variants predominantly belonging to the class Clostridia and the genus *Clostridium* (Fig. 5a). Notably, *Prevotella copri_A* emerged as one of the top taxa, harboring 1,776 significant variants across 573 genes, whereas *Bacteroides uniformis* exhibited only 21 significant variants in 11 genes (Supplementary Table 14).

To understand the functional consequences of these associations, we examined the KEGG modules enriched in the affected genes. We found that different taxa were enriched for distinct functions: *Prevotella* variants were associated with amino acid and KDO2-lipid A biosynthesis, *Clostridium* variants with glucose metabolism, and *Blautia* variants with cobalamin and siroheme biosynthesis (Fig. 5b).

Given that approximately two-thirds of individuals in the OA cohort were infected with helminths, we next asked whether helminth infection might partially explain the observed strain-level differences between the OA and KL cohorts. To address this, we performed a separate mGWAS comparing helminth-infected and uninfected individuals within the OA cohort. While *Clostridia* remained the top class with the most significant hits, the top associated genus shifted to *Blautia*, with over 1,400 altered genes. In contrast, *Clostridium* showed a four-fold reduction in the number of hits, suggesting that *Clostridium* is more strongly linked to urbanization, whereas *Blautia* is more associated with helminth infection (Fig. 5c).

Functional profiling further supported this interpretation. Genes in *Blautia* identified from both comparisons showed similar functions, indicating that its enrichment in the OA vs. KL analysis is largely driven by helminth infection (Fig. 5b) (Supplementary Table 15). By contrast, *Prevotella* had only 36 genes associated with helminth infection (Fig. 5c) and no enriched functional modules (Fig. 5b), suggesting that its strain-level variation is more reflective of lifestyle differences rather than helminth-infection status.

### Strain-level variation correlates with host cytokine levels

**Extended Data Fig. 5:**
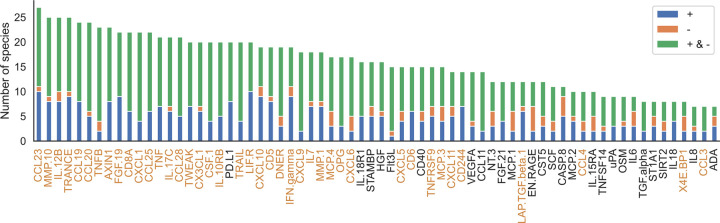
Cytokines ranked by the number of microbial species with correlated strain-level variations. Microbial species are categorized based on the direction of correlations of their genetic variants with cytokine levels: species with only variants showing positive correlations (+), species with only variants showing negative correlations (−), or species with variants showing both positive and negative correlations (+ & −)with certain cytokines. Cytokines highlighted in brown are those significantly correlated with Prevotella strain-level variants.

**Fig. 6:**
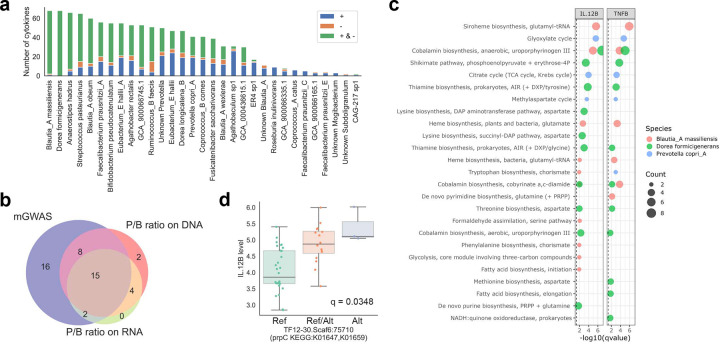
Strain-level variations in the gut microbiome correlates with host cytokine and chemokine levels. (**a**) Microbial species ranked by the number of cytokines significantly correlated with their strain-level variants. Cytokines are grouped by correlation direction: exclusively positive (+), exclusively negative (−), or both positive and negative for at least one strain-level variant of the species (+ & −). (**b**) Overlap between cytokines correlated with *Prevotella* strain-level variants (“mGWAS”) and those correlated with the *Prevotella:Bacteroides* abundance ratio in metagenomic (“P/B ratio on DNA”) and metatranscriptomic (“P/B ratio on RNA”) data. (**c**) KEGG modules enriched among genes containing cytokine-correlated variants for representative species and cytokines. Dot size indicates the number of genes per module; vertical lines indicate statistical significance after correction for multiple testing. The top ten enriched modules per species are shown. (**d**) Example of a *Prevotella copri_A* variant in the prpC gene significantly correlated with IL-12B levels. Samples were grouped by genotype: only reference allele (“Ref”), only alternative allele (“Alt”), or both alleles (“Ref/Alt”). Q-value was calculated using Spearman correlation and adjusted for multiple testing with the Benjamini–Hochberg method.

To explore the relationship between microbial strain-level variation and host immunity, we assessed microbial variants associated with host cytokine levels. We identified 36 microbial species with at least one variant significantly correlated with at least one cytokine (Fig. 6a and Supplementary Table 16), resulting in 68 different cytokines with at least one strain-level association (Extended Data Fig. 5). Among the top taxa were *Blautia_A massiliensis* and *Dorea formicigenerans*, both of which were also enriched in helminth-infected individuals (Fig. 5c), suggesting that strain-level variation in these taxa may modulate immune responses to helminths.

Additionally, *Prevotella copri_A* harbored variants associated with 41 cytokines, while no significant associations were detected for *Bacteroides*. Among the 41 cytokines linked to *P. copri_A* strain-level variants, 25 also correlated with the *Prevotella/Bacteroides* abundance ratio in either DNA or RNA data or both, whereas 16 were uniquely associated with strain-level variation and not abundance (Fig. 6b). In contrast, only 6 cytokines were correlated exclusively with abundance ratios, indicating that strain-level differences may have a stronger influence on host-microbiome interactions than taxonomic abundance alone.

To gain insight into functional consequences, we analyzed KEGG modules enriched among cytokine-correlated variants in *P. copri_A*, *B. massiliensis*, and *D. formicigenerans*. Variants in *B. massiliensis* were enriched in siroheme and cobalamin biosynthesis, consistent with modules observed in helminth-associated comparisons. *D. formicigenerans* similarly showed enrichment in cobalamin biosynthesis, suggesting a potential mechanistic link between cobalamin metabolism and immune modulation during helminth infection (Fig. 6c). For *Prevotella*, cytokine-correlated variants were enriched in the glyoxylate cycle. For example, a variant in the *prpC* gene showed a significant correlation with IL-12B levels (Fig. 6d). Together, these results underscore the functional relevance of strain-level variation in shaping host immune responses.

## Discussion

By better understanding gut microbiome function in the Orang Asli, we gain insights into how urbanization and traditional lifestyles shape microbial activity and host interactions. In this study, we integrated newly generated metatranscriptomic data from 66 OA and 30 urban KL samples with our previously published metagenomic datasets comprising 650 fecal samples, including 351 from Indigenous OA and 56 from urban KL individuals in Malaysia^3^. To our knowledge, this is the first stool metatranscriptomic survey of Southeast Asian Indigenous populations, providing novel insights into the transcriptional activity of key gut bacteria such as *Prevotella* and *Bacteroides* and their interactions with host immune responses. Furthermore, we present the first analysis of microbial gene expression of these taxa in the context of helminth infections, which are endemic among rural OA communities. By applying a mGWAS method, we identified microbial SNPs linked to lifestyles and host immune markers, supporting the hypothesis that genetic diversity within bacterial species contributes to divergent functional and immunological roles. These findings provide an integrated view of host-microbiome interactions associated with lifestyle modernization and endemic chronic parasitic exposure.

A central finding was the differential transcriptional activity of *Prevotella* and *Bacteroides* across lifestyle groups (Fig. 3b and c). These genera have emerged as key microbial indicators of lifestyle transitions across various cohorts^37,39,41–47^, including our own 16S rRNA-based comparison of Malaysian OA and urban populations^27,48^. While these studies primarily focused on taxonomic differences, our metatranscriptomic analysis revealed that the well-documented inverse relationship between these genera extends beyond mere abundance to involve fundamental differences in metabolic engagement. Lifestyle appears to shape not only which microbes are present, but also which are actively functioning. We found all *Prevotella* species were more abundant and transcriptionally active in OA, while all *Bacteroides* species were more abundant and active in KL (Extended data Fig. 3a), indicating sustained functional engagement. *Prevotella* species, including *P. copri* and previously uncharacterized species, showed broad activity in OA. This likely reflects the fiber-rich, diverse plant diets of traditional communities, which provide multiple opportunities for different *Prevotella* species to contribute to carbohydrate breakdown^54,70^. These findings emphasize that lifestyle-associated microbial differences are not only compositional but also functionally decoupled, with some species present in abundance yet transcriptionally silent. This highlights the importance of incorporating functional profiling for understanding gut microbiome dynamics.

The inverse transcriptional activity of *Prevotella* and *Bacteroides* in this study suggests that the balance between these genera may play a functional role in modulating gut immune homeostasis. We observed that higher *Prevotella/Bacteroides* ratios correlated with increased levels of mucosal immune mediators such as CCL20, CCL25, and CCL28, which regulate immune cell trafficking (Fig. 4a and b), including Th17 cells, CCR9^+^ T cells, and IgA-secreting plasma cells^71–73^. Epithelial cytokines like IL17C also showed elevation, suggesting enhanced barrier function^74^. Our findings are supported by accumulating evidence that *Prevotella* species, particularly at mucosal sites, can stimulate epithelial and antigen-presenting cells to produce pro-inflammatory cytokines, thereby promoting Th17 polarization and neutrophil recruitment^75,76^. Conversely, the *Bacteroides* species identified in our urban KL cohort such as *B. stercoris*, *B. thetaiotaomicron* and *B. uniformis* have showed metabolic benefits and potential immune-regulatory properties, species like *B. caccae* are primarily recognized as commensals with occasional pathogenic potential and remain relatively understudied^77–84^. Their presence may represent a distinct subset of *Bacteroides* that contributes to immune regulation and epithelial homeostasis, potentially counterbalancing the immune-stimulatory effects of *Prevotella* to promote a more tolerogenic mucosal environment.

These mechanistic patterns align with our observation that higher *Prevotella/Bacteroides* ratios correspond to coordinated upregulation of mucosal immune markers. Our findings contribute to ongoing debates over beneficial versus detrimental roles of *P. copri* in human health^85,86^, suggesting that *Prevotella*-dominated communities may drive heightened state of mucosal immune activation with context-dependent effect on host physiology. Consistent with this, we observed significantly elevated levels of a broader array of inflammatory markers in the OA cohort compared to urban KL residents (Fig. 4d), reflecting a more activated immune baseline. This immune priming may be adaptive in traditional lifestyle environments characterized by persistent microbial exposures, such as helminths, enteric viruses, or environmental pathogens, by enhancing mucosal surveillance and barrier integrity. Conversely, in urban or low-pathogen settings, such activation may be maladaptive, potentially predisposing individuals to chronic inflammation or immunopathology. Thus, the OA cohort provides an illustrative example supporting the context-dependent nature of *Prevotella*-host interactions. Future research is needed to elucidate causality and explore how microbial function modulates immune outcomes in diverse environmental contexts.

To explore the genetic basis underlying these species-specific functional responses, we examined strain-level genomic diversity and its associations with host immune parameters. The extensive strain-level diversity observed in *Prevotella copri_A*, with over 1,700 significant variants distinguishing OA from KL individuals, far exceeds that observed in *Bacteroides uniformis*. This pattern suggests that certain gut microbes serve as evolutionary nodes for functional diversification. These variants, not overall abundance, were significantly associated with host cytokine and chemokine levels (Fig. 6b). This finding helps reconcile conflicting reports of both pro- and anti-inflammatory effects of *P. copri*^85,86^, suggesting that its immunological role is strain dependent. For example, as shown in our study, variants in the glyoxylate cycle gene *prpC* were significantly associated with IL12B expression (Fig. 6d), implicating them in Th1 and Th17 modulation^87,88^. Additionally, *P. copri_A* variants were associated with KDO2-lipid A biosynthesis (Fig. 5b), a highly conserved component of lipopolysaccharide (LPS) known to trigger innate immune responses via TLR4^89^. These genetic differences may influence structural or regulatory aspects of lipid A production, potentially modulating its immunostimulatory capacity and contributing to the heightened but non-pathological immune tone observed in OA individuals.

Importantly, different taxa exhibited contrasting responses to urbanization versus helminth infection. *Blautia_A massiliensis and Blautia_A obeum* variants were consistently enriched for similar metabolic functions (cobalamin and siroheme biosynthesis) regardless of comparison type (OA vs. KL or helminth infection status) (Fig. 5b). In contrast, *P.copri_A* variants showed minimal helminth-specific associations. This suggests that these taxa follow distinct ecological strategies. *Blautia* species may be specialized responders to parasitic infections, while *P.copri_A* appears more sensitive to broader ecological transitions, such as lifestyle modernization. Collectively, these findings highlight that current abundance-based microbiome analyses may overlook key functional variations and support a shift toward strain-resolved approaches that better capture microevolutionary adaptations and their immunological consequences.

Building on these findings, we also examined how helminth infection, a common exposure in OA modulates gut microbial function. Helminth infections have been shown to alter gut microbial composition across diverse settings^3,90,91^, including our prior work in OA revealed increased microbial diversity and village-specific compositional shifts^3^. Elevated *Prevotella* abundance have similarly been observed in our and other parasitic-endemic population^27,92–96^. While overall *Prevotella* genus activity was unchanged, specific *Prevotella* species showed elevated transcription in infected individuals (Fig. 3f, h, i and Extended Fig. 4), suggesting species-specific functional responses to helminth exposure. In contrast, *Bacteroides* species active in urban KL residents (*B. uniformis*, *B. caccae*, *B. stercoris*) showed higher activity in our helminth-uninfected individuals, consistent with their abundant in parasitic uninfected individuals^3,92,93,97^. As discussed above, these species may contribute to a more tolerogenic mucosal environment, reinforcing our proposed model in which *Bacteroides* and *Prevotella* play opposing, context-dependent effects on immune regulation.

We identified 307 prokaryotic species specific to Malaysian individuals, absent from both the UHGG^30^ and KIJ^33^ catalogs (Fig. 1b, c and d). Approximately half belong to the class Clostridia, indicating a strong enrichment and previously underexplored diversity of Clostridia in this populations. The majority were assembled from OA individuals. These findings, alongside with our prior evidence of helminth-associated increases in Clostridiales and reduced inflammatory bowel disease symptoms, support the idea that helminths promote Clostridia diversity linked to immune regulation^27^. This is further confirmed by Clostridia species isolated from helminth-infected OA individual, which have been implicated in both supporting helminth lifecycle^98^ and exerting anti-inflammatory effects^99^. Together, these findings highlight OA-specific Clostridia as potential mediators of co-evolved host–microbiome relationships shaped by helminth infection.

Among the newly identified Malaysian Clostridia, we found a significant number of putative species under *HGM13006* genus, enriched in starch and sucrose metabolism pathways (Fig. 2a and b). To date, the genus has only been reported of human samples from China (BioSample accession: SAMEA5279163). In our study, it was exclusively assembled from the OA population. These metabolic features likely reflect adaptation to traditional high-starch diets among OA communities, including rice and root crops, which are usually foraged and cultivated locally. We also identified Malaysian-specific Clostridia species belonging to the *Ruminococcus_D* clade (Fig. 2c and d). *Ruminococcus* is a polyphyletic genus that includes members of multiple families with distinct profiles of carbohydrate-active enzyme genes^100^. While *R. gnavus and R. torgues* (family: Lachnospiraceae) have been linked to gut inflammation and *R. bromii* (Acutalibacteraceae or Oscillospiraceae) is associated with probiotic potential^101–106^, relatively little is known about *Ruminococcus_D* (Ruminococcaceae). Phylogenetic analysis places our taxa near *Ruminococcus_D bicirculans*, a species capable to degrade plant polysaccharides and negative associated with tryptophan levels in colorectal cancer^107,108^. Although *R. bicirculans* has predominantly been reported in westernized populations^100^, our recovery of related taxa from Malaysian individuals suggests possible population-specific enrichment. Functional enrichment analysis revealed that Malaysian *Ruminococcus_D* species are enriched in flagellar assembly and chemotaxis pathways, suggesting motility and environmental sensing capabilities. These features may facilitate spatial localization within the gut, similar to *Roseburia* from the same family, which use flagella-mediated chemotaxis to navigate toward mucin and short-chain fatty acids^109^. Therefore, future efforts to culture and experimentally characterize both *HGM13006* and *Ruminococcus_D* are crucial to unravel their ecological roles, host interactions, and potential therapeutic applications.

While our study provides new insights into the gut microbial activity and genetic variation in the microbiome of an Indigenous population in Southeast Asia, several limitations should be acknowledged. First, our focus on OA communities in Malaysia may limit the generalizability of findings to other regions. Future studies should include a broader range of Indigenous populations from various geographic locations to access whether the observed microbial functions are consistent across different cultural and environmental contexts. Second, our genomic analysis relies on existing reference databases, which may not include poorly characterized or population-specific microbial species. This could result in underrepresentation of key taxa and limit the detection of relevant SNPs within microbial species or functional pathways. Expanding region-specific reference genomes will be essential to improve the accuracy of microbial variant detection in underrepresented populations.

In conclusion, this study contributes a comprehensive functional perspective on the gut microbiome of Southeast Asian Indigenous populations, addressing a significant gap in microbiome research. Our findings establish a mechanistic bridge between microbial genetics and host immunity, moving beyond taxonomic profiles to functional insights. Through the integration of metatranscriptomic and metagenomic data, we identified distinct transcriptional profiles of key microbial taxa (*Prevotella* and *Bacteroides*) associated with traditional lifestyle, helminth exposure, and mucosal immune activation. The association of *Prevotella*-linked SNPs with the expression of mucosal cytokines highlights the influence of microbial genomic diversity in shaping host immune responses. Additionally, we identify potentially novel *Clostridia* species unique to the Malaysian cohort, reflecting possible adaptations to dietary habits and roles in immune regulation. These insights emphasize the importance of including diverse human populations in microbiome research to better capture the full spectrum of functional and ecological variation for human health.

## Methods:

### Ethics approval and community engagement

This study builds on two decades of research involving OA communities, in collaboration with government officials from the Malaysia Department of Orang Asli Development (JAKOA) under the Ministry of Rural and Regional Development, Universiti Malaya Medical Centre (UMMC), and the Faculty of Medicine, Universiti Malaya. Through years of collaboration and engagement with the relevant government ministry, we have fostered trust with OA communities while promoting health awareness and addressing health disparities. Our initial research with OA communities focused on epidemiological studies of infectious diseases and later expanded to microbiome research (gut and skin), particularly in relation to helminth infections and skin diseases. This study extends our previous work by assembling genome datasets and enhancing existing gut microbiome databases, ultimately creating a more comprehensive gut microbiome resource that includes data from Southeast Asia, with a focus on Malaysian Indigenous communities.

This study was approved by the UMMC Medical Ethics Committee of Universiti Malaya Medical Centre (MREC) (Reference No.: 2017925–5593), National Medical Research Register (NMRR), Ministry of Health, Malaysia (Reference No.: NMRR-17-3055-37252), New York University Institutional Review Board (Reference No: i17–01068), in compliance with guidelines for human subject protection, including the Declaration of Helsinki and Good Clinical Practice. Additional approval was obtained from the Department of Orang Asli Development (JAKOA in the national language) (Reference No.: JAKOA/pp.30.052Jld13 and JAKOA/pp.30.052Jld14) to conduct the research at OA villages. JAKOA is a government agency responsible for overseeing the welfare and development of the OA) communities. Permission will also be obtained from the village chieftain, reflecting our commitment to proper governance, community protection, and respect.

Prior to the commencement of the study, multiple discussions were held with JAKOA officers, village chieftains, and community members to explain the study’s purpose. These conversations extended beyond the research scope, covering broader health issues, healthcare access, cultural concerns, and community priorities. Additionally, the study schedule was coordinated with the respective village chieftains to ensure smooth participation and community engagement. The continuous input and feedback from OA communities have been essential in refining our research focus, deepening scientific understanding of OA health challenges, and strengthening trust over the years.

During the research, an oral briefing on the study’s purpose and procedures was provided to participants through both community gatherings in a hall and a door-to-door approach. This ensured that all villagers had access to the study and fully understood its objectives. Communication, including briefings, interviews, and health education, was conducted in Malay, the national language of Malaysia. For those who did not understand Malay, a local translator facilitated translation into their native language to ensure clear comprehension. In addition to research, simple health education on disease prevention was provided as part of the study. The study findings will be shared with enrolled participants and relevant stakeholders, including healthcare officials and authorities, to support collective efforts in addressing the OA community’s concerns and improving long-term health outcomes. To show appreciation, enrolled participants were provided with food and drinks.

### Study population and sample collection

This study involved 351 OA individuals from five villages: (1) Rasau village (Perak state); (2) Judah village (Selangor state); (3) Sepat village (Selangor state); (4) Bangkong village (Selangor state); and (5) Legong village (Kedah state), along with 56 urban citizens from Kuala Lumpur, Malaysia, as reported in a previous study^3^. A total of 650 stool samples were collected for metagenomics analysis as previously described^3^, with an additional subset of 96 stool samples were subjected to metatranscriptomic analysis in this study. The OA cohort consisted of both helminth-infected and uninfected individuals, and the study was conducted in cross-sectional and longitudinal phases^3^. In the longitudinal phase, consented OA subjects (both helminth-infected and uninfected) received 400 mg of albendazole daily for three consecutive days^3^. Due to the COVID-19 pandemic, the study was limited to four villages to assess the effects of anthelminthic treatment at 21 day and 42 day post-treatment^3^. Detailed participant sample collection numbers are outlined in Fig. 1a.

Additionally, 314 blood samples were collected. Of these, 187 blood samples from participants in Rasau and Judah villages were outsourced to two certified laboratories: BP Healthcare Sdn Bhd (Registration No.: 1357064W / 202001000745) and Quantum Diagnostic Sdn Bhd (Registration No.: 0941541V / 201101013401) for blood testing. The testing panel included complete blood counts, liver function tests, renal function tests, lipid profiles, glucose levels and trace element analysis. The results were shown in Supplementary Table 17. Of the 187 samples analyzed, only the pre-anthelminthic samples (n=107) with matching metagenomic data were included in the *Prevotella*/*Bacteroides* association analysis. Meanwhile, 127 blood samples from participants in Sepat, Bangkong, and Judah villages, collected on Whatman protein saver cards, were sent to Olink Proteomics, Uppsala, Sweden, for the analysis of 92 inflammation-related proteins using Olink’s proximity extension assay technology. After filtering out those below the determined threshold, sixty-eight inflammatory-related proteins (Supplementary Table 18) were proceeded with statistical analysis.

Prior to enrollment, subjects were screened for eligibility based on the following criteria: (1) aged between 4 and 85 years and (2) a resident of the respective village. Exclusion criteria included (1) pregnancy, (2) breastfeeding, and (3) the presence or suspicion of any clinically significant illness. Written consent was obtained from all subjects aged 18 and above. For children under 18, consent was provided by a parent or guardian, and assent was obtained from those aged 7 to 17 years.

### Sample processing and analysis

The methodology for sample preprocessing and helminth detection has been outlined in our previous study^3^. As for metatranscriptomics, the aliquoted stool samples were preserved in RNA*later* (Invitrogen, AM7024) and stored in −80°C freezer until processing. Prior to RNA extraction, each sample were vortexed, centrifuged at 16,000g at 4°C for 10 minutes to remove the RNA*later*. Subsequently, the stool pellets were washed with RNase-free water, vortexed and centrifuged again for 10 mins. RNA extraction was performed on the homogenized samples using RNeasy PowerMicrobiome Kit (Qiagen, 26000–50). The resulting pellet (~ 250mg) was first homogenized using a tissue lyzer in a bead tube with Solution PM1–β-ME, followed by manufacture’s protocol for extraction. Then, the concentration and integrity of the extracted RNA were assessed using High Sensitivity RNA ScreenTape assays on the Agilent 4200 Tapestation System (Agilent, 5067–5579, 5067–5581 & 5067–5580). Subsequently, 100 ng of total RNA from each of the 96 samples was used to prepare RNA-Seq libraries according to the Illumina Stranded Total RNA Prep with Ribo-Zero Plus protocol (Illumina, 1000000124514-v02) at the NYU Langone Genome Technology Center. The prepared metatranscriptomic libraries were sequenced on the Illumina NovaSeq 6000 S2 platform, generating paired end reads of 50 base pairs in length for downstream bioinformatic analyses.

### MAGs assembly

Sequence quality was assessed and visualized using FastQC v0.12.1^110^ and multiQC v1.20^111^. Raw sequencing data (13,271,068,192 reads) were trimmed to remove adapter segments and low-quality ends, then filtered to remove shorter reads (<50 bp) using Trimmomatic v0.36^112^ (v0.36) with default settings. To remove human contamination, the trimmed reads were mapped to the human reference genome (hg38) using BMTagger v3.10^113^. After filtering, a total of 6,623,619,570 paired ends microbial quality-filtered reads were retained, with 2,471,700 to 35,535,952 microbial reads per sample (mean = 20,151,612 reads per sample, SD = 3,796,408). The pre-processed reads from each sample were assembled separately using the MetaWRAP v1.3.2^114^ (v1.3.2) assembly module. This module first assembles all reads with metaSPAdes v3.15.5^115^ (v3.15.5) first then assembles remaining unassembled reads with MEGAHIT v1.2.9^116^ (v1.2.9), and finally combines contig outputs (minimum 1 kb length) from both assemblers. This resulted in a total of 16,560,789 assembled contigs, with 5,193 to 52,356 contigs per sample (mean = 25,557 contigs per sample, SD = 7,772). Assembly quality was assessed using QUAST v5.0.2^117^. The largest contig assembled per sample ranged from 148.12 Kbp to 1,730.15 Kbp.

MAGs were built from metagenomes following a previously published workflow^118^. In brief, the assembled genomes were binned using three different algorithms: MetaBAT2 (machine learning-based, n = 27,331), MaxBin2 (expectation-maximization-based, n = 35,910), and CONCOCT (Gaussian mixture model-based, n = 51,521), as implemented in the MetaWRAP modules^114^. The quality of the binned genomes was further refined using the bin_refinement function of MetaWRAP^114^, with a completeness threshold of 0 and a contamination threshold of 100. This process resulted in 55,415 consolidated metagenomics bins, which were further quality-checked using both checkM^119^ and checkM2^120^. Bins that met a completeness threshold of 50% and a contamination threshold of less than 10% were retained, yielding 19,851 bins. Chimeric checking was performed via GUNC v1.0.551^121^. The non-chimeric, refined genomes (n = 15,867) were then combined with genomes from the UHGG (n = 4,644)^30^ and KIJ (n = 29,082)^33^ for dereplication using dRep^122^. The dereplication parameters were set for an average nucleotide identity (ANI) of 95% and minimum overlap of 30%. MAGs originating from clusters containing only Malaysian MAGs were classified as “Malaysia MAGs”. The dereplicated MAGs (n = 5,355) were assigned taxonomy using GTDB-Tk v2.3.2^123^, and phylogenetic trees were constructed based on alignments generated by GTDB with IQ-TREE v2.2.0.5^124^.

### Building of the Malaysia MAGs catalog

To construct the Malaysian MAG catalog, the quality of the non-chimeric and refined metagenome-assembled genomes (MAGs, n = 15,867) was evaluated. MAGs with a completeness of at least 90.0%, contamination of <5%, the presence of 5S, 16S, and 23S rRNA, and at least 18 unique tRNAs were classified as high-quality (n = 439). MAGs with a completeness of at least 90.0% and contamination of <5% were categorized as near-complete (n = 7,096), while MAGs with a completeness of at least 50.0% and contamination of <10% were categorized as medium-quality (n = 8,332), following the criteria established by Bowers et al. (2017) (Supplementary Table 1). The presence or absence of large and small subunit rRNA genes were assessed using Infernal v1.1.15^125^ and the number of tRNA genes were assessed using tRNA-scan-SE v2.0.9^126^. These MAGs were further dereplicated, resulting in a Malaysian MAG catalog comprising 1,179 MAGs (Extended Data Fig. 1).

### Metatranscriptomic read processing

Sequence quality was assessed and visualized using FastQC v0.11.8^110^ and multiQC v1.20^111^. Raw sequencing data (~8.13 billion reads) were trimmed to remove adapter sequences and low-quality bases using Trimmomatic v0.36^112^ with default parameters. To remove human contamination, the trimmed reads were mapped to the human reference genome (hg38) using Bowtie2 v2.3.5.1^127^. After filtering, a total of 759 million paired-end microbial quality-filtered reads were retained. To enrich for messenger RNA, non-human reads were filtered against rRNA databases using SortMeRNA v4.3.4^128^, yielding 738 million non-rRNA (mRNA-enriched) reads for downstream analysis (mean = 7,687,637 reads per samples, SD = 5,030,740).

### Reads mapping

The Malaysian MAG catalog (5,355 representative MAGs) were incorporated into the Kraken 2 RefSeq Standard PlusPFP v2.1.2 database using the kraken2-build function to create a custom database^129^.Taxonomic classification was then performed using Kraken2 v2.1.2^129^ with default settings and the custom database. Bracken v2.8.0^130^ was then used to estimate abundance at the species level. The resulting compositional data were output to BIOM file format via Kraken-biom v1.2.0^131^. This step was done on both metagenomic and metatranscriptomic reads. Finally, the BIOM files, together with metadata from the questionnaire and helminth infection status, were merged to form a phyloseq object using the phyloseq package^132^ in R v4.1.3. The phyloseq object formulated earlier was further processed which, taxa with fewer than 100 reads within a sample were eliminated. Subsequently, the abundance table was normalized via multiple rarefactions (1000 times) using the phyloseq_mult_raref function of the metagmisc package^133^, downsampling to 6,006,298 (DNA) and 649,505 (RNA) reads per sample (the minimum sample read count) before further analysis.

### Identification of functional pathways that are enriched in Malaysia *HGM13006* and *Ruminococcus_D*

The *HGM13006* and *Rumonococcus_D* MAGs were annotated using Prokka v1.4.6^134^. The resulting annotated genes (in *.faa format) were mapped to their respective KEGG IDs using eggNOG-mapper v2^135^. Then the Malaysia and known MAGs within these two genera were clustered separately using ClusterProfiler^135^ for comparison of functional pathway enrichment.

### Microbial genome-wide association analysis (mGWAS) from metagenomes

We used GT-Pro v1.0.1^66^ to genotype 881 microbial species from gut metagenomes using default parameters. Variants supported by at least two read counts were retained.

We performed two mGWAS comparisons: (1) OA vs. KL, and (2) helminth-infected vs. uninfected individuals within the OA cohort. For each species, each variant was tested for enrichment using Fisher’s exact test by comparing samples carrying only the reference allele to those with at least one read matching the alternative allele. To assess associations with host cytokines, we performed Spearman correlation between variant genotypes and cytokine levels. Genotypes were encoded as follows: 1 for all reads matching the alternative allele, 0 for reads matching both alleles, and −1 for all reads matching the reference allele. All p-values were adjusted for multiple testing using the Benjamini–Hochberg method. Variants with adjusted p-values < 0.05 were considered significant.

To evaluate functional consequences, significant variants were mapped to genes annotated with Prokka v1.14.6^134^ on the GT-Pro reference genomes. Genes were assigned KEGG orthologs using eggNOG-mapper v2.1.6^135^. Enrichment of KEGG modules among genes harboring significant variants was performed using the clusterProfiler R package^136^. Although both enrichment and correlation analyses indicate the directionality of the alternative allele, we acknowledge that this directionality is relative and depends on the choice of reference genome. Therefore, all significant variants were pooled prior to functional analysis.

### Quantification & statistical analysis

The phyloseq object was normalized via multiple rarefactions (1000 times) using the phyloseq_mult_raref function of the metagmisc package^133^, downsampling to 1,481,547 reads per sample (the minimum sample read count) before further analysis.

All statistical analyses and data visualizations were conducted in R v4.1.3. Linear Regression analysis to find the association between *Prevotella*/*Bacteroides* ratio with cytokines and chemokines and blood parameters was done using lm() function of the stats package in R^137^. Furthermore, the phyloseq objects were converted to dataframe using psmelt() function of phyloseq package^132^. Data visualization was performed using either the ggpubr^138^ or ggplot2^139^ packages of R. Microbiome Multivariable Association with Linear Models 2 (MaAsLin2)^69^ was used to identify taxa differentially abundant between OA and KL cohorts, with age and gender included as fixed effects. For comparisons between helminth-infected and non-infected individuals, age, gender, and village location were included as fixed effects. Results were filtered to retain only taxa with a minimum prevalence of 15%, and only MAGs passing validation thresholds using CoverM^140^ (≥20% genome coverage at the 90th percentile) were considered for downstream interpretation. P-values were adjusted using the Benjamini-Hochberg method.

To compare the chemokines and cytokines between groups, statistical significance was assessed using the Wilcoxon rank-sum test implemented in the rstatix package^141^. P-values were adjusted for multiple comparisons using the Benjamini–Hochberg method. Significantly different variables were selected for radar plots generated with the *fmsb* package in R^142^, using log-normalized median values.

## Extended Data

## Supplementary Material

Supplementary Files

This is a list of supplementary files associated with this preprint. Click to download.

- SupplTables080325.xlsx

## Data Availability

The metatranscriptomic raw reads has been deposited on the NCBI Sequence Read Archive with the Bioproject No. PRJNA1331796 and BioSample accession No. 45911716–45911811 and will be available upon published. The Malaysian MAGs will be deposited in the https://research.nhgri.nih.gov/projects/ upon publication. The shotgun metagenomic data have been deposited previously^3^ on the NCBI Sequence Read Archive with the BioProject No. PRJNA797994 and BioSample accession No. SAMN25042866–25043515. In addition, the data have also been uploaded to MicrobiomeDB (microbiomedb.org) under named “Malaysia helminth study”. Blood test and Olink Protein results are presented in Supplementary Table 17 and Table 18, respectively.

## Code availability

All code used for the analysis in this study will be made available at Github repository https://github.com/idss-niaid upon publication.
